# Retraction: Comparison of Steroid-Free Immunosuppression and Standard Immunosuppression for Liver Transplant Patients with Hepatocellular Carcinoma

**DOI:** 10.1371/journal.pone.0222257

**Published:** 2019-09-04

**Authors:** The PLOS ONE Editors

Concerns have been raised that the transplants performed in the local context at the time of procedures reported in this article [1] may have involved organs/tissues procured from prisoners [2].

Details as to the donor sources and methods of obtaining informed consent from donors were not reported in [1], and the authors did not reply to journal queries seeking to clarify these issues and the cause(s) of donor death. International ethics standards call for transparency in organ donor and transplantation programs and clear informed consent procedures including considerations to ensure that donors are not subject to coercion.

In addition, the authors did not provide documentation when requested by the journal to confirm that the study had institutional ethics approval, and they did not respond to inquiries about the availability of underlying data supporting this study.

Owing to the lack of documentation to demonstrate this study had prospective ethical approval, insufficient reporting, unresolved concerns around the source of transplanted organs and whether they included organs from prisoners, and in compliance with international ethical standards for organ/tissue donation and transplantation, the *PLOS ONE* Editors retract this article.

The authors did not respond to the retraction notification.
